# The Effect of the Addition of Hazelnut or Walnut Flour on the Rheological Characteristics of Wheat Dough

**DOI:** 10.3390/ma15030782

**Published:** 2022-01-20

**Authors:** Karolina Pycia, Lesław Juszczak

**Affiliations:** 1 Department Food Technology and Human Nutrition, Institute of Food Technology, College of Natural Science, University of Rzeszow, Zelwerowicza 4 St., 35-601 Rzeszow, Poland; 2 Department of Food Analysis and Evaluation of Food Quality, University of Agriculture in Krakow, Balicka 122, 30-149 Krakow, Poland; rrjuszcz@cyf-kr.edu.pl

**Keywords:** wheat dough, nuts, starch, rheological properties, water absorption

## Abstract

The aim of this study was to evaluate the effect of the addition of hazelnut or walnut flour on the rheological properties of flour and wheat dough (WD). The research material was a system in which wheat flour was replaced with flour based on hazelnuts (HF) or walnuts (WF), i.e., nut flour, in the amounts of 5%, 10% and 15% (WDH, WDW). As a part of the research methodology, we analysed the wet gluten content, and farinographic and extensographic analyses of the dough were performed. Sweep frequency, creep and recovery tests were used to assess the viscoelastic properties of the tested doughs. It was found that the doughs with the addition of walnuts were characterized by different rheological properties compared to the control sample. The systems in which wheat flour was replaced with nuts were characterized by lower water absorption, and this parameter decreased as the share of nuts in the system increased. The mean value of this parameter for WDH was 48.6%, and in the case of WDW it was 47.9%. The development times of WDH and WDW doughs were longer compared to the control, but they decreased as the addition of nut flour was increased. The WDH doughs were characterized by the lowest stability and the highest degree of softening among the examined doughs. It was shown that the addition of nut flour reduced the values of the storage (*G′*) and loss (*G″*) modules characterizing the tested doughs, while in each case the *G′* value was greater than the *G″* value, which proves the advantage of the elastic properties. The creep and recovery tests showed that the nut dough was more susceptible to deformation compared to the control, which indicates that the presence of nut flour weakens the formation of the gluten network forming the dough structure, and makes it more susceptible to stress.

## 1. Introduction

The main component of wheat flour resulting from the grinding of wheat grains is starch. This biopolymer plays the most important role in shaping the rheological properties of the dough [1]. Nevertheless, the remaining ingredients in the flour also influence the rheological properties of the dough. In addition to the starch content, the type and amount of protein, fat and fiber contents are also important. Among all cereal flours, only wheat flour, when mixed with water, has the ability to form a three-dimensional, viscoelastic dough [2]. Macroscopically, the dough is a homogeneous mixture of starch, protein, fat, salt, yeast and other ingredients. In terms of rheology, it is a system consisting of two dispersed phases, i.e., gas bubbles embedded in a starch–gluten matrix [3]. With optimal mixing, the dough is fully hydrated and has the highest flexibility [4]. This property largely depends on the presence of non-protein components that may hinder the formation of an elastic system. Devices such as a farinograph, an extensograph or a mixograph are used in the analysis of the rheological properties of the dough because they provide the practical information necessary for the proper planning of the bread production process. Basic rheological studies in the area of linear viscoelasticity provide information on the structure of the dough and the functions of its ingredients [4]. In general, knowledge of the rheological properties of bread dough is important in order to characterize and determine the efficiency of the dough during its processing, and to shape the appropriate quality of the final product, which is bread. This knowledge is particularly important for practitioners because it allows them to determine the behavior of the dough during the mechanical processing, fermentation process and baking of the dough [5]. As a part of the daily diet of most consumers, bread is also useful in the process of fortified food. As a result, people’s nutritional status can be improved by increasing the supply of nutrients, vitamins, minerals and bioactive substances in products that are part of the daily diet of most consumers. Therefore, bread is a good carrier of these substances. From the point of view of the increasingly common process of enriching bread with ingredients of plant or animal origin, it is important to know not only their impact on the quality of the finished product but also the rheological properties of the bread dough, which are likely to change under the effect of these ingredients. Nuts are an innovative plant material used in food fortification. According to Gómez et al. [6] nuts are a rich source of fat, protein, fiber and minerals. These ingredients not only help to improve the health-promoting values of the bread, but also affect its nutritional value and the technological features of the dough. The studies conducted so far indicate the possibility of using walnuts and hazelnuts and products based on them, such as oil and cake [7]—to enrich wheat bread. However, it should be remembered that replacing a part of the flour with another ingredient, such as ground nuts, which do not contain gluten and are rich in fat, probably affects the rheological properties of the dough based on the wheat flour–nut system. The literature on the subject lacks data on this subject; therefore, the aim of the study is to fill this gap and evaluate the effect of the addition of ground, dry walnut or hazelnut seeds on the rheological and textural properties of wheat dough.

## 2. Materials and Methods

### 2.1. Materials 

The research material was a system of type 650 universal wheat flour (Gdańskie Młyny, Gdańsk, Poland) with the addition of hazelnut flour (HF) and walnut flour (WF) from the 2020 harvest, from which the dough was made (WD). In the developed mixtures, wheat flour was replaced with hazelnut flour and walnut flour in the amount of 5% (WDH5%, WDW5%), 10% (WDH10%, WDW10%) and 15% (WDH15%, WDW15%). The control was wheat dough without any addition of nut flour (control).

The tested wheat flour contained 1.1 ± 0.1% d.m. fat and 2.5 ± 0.1% d.m. crude fiber. The addition of hazelnut flour in the amount of 5, 10 or 15% caused a significant increase in the fat content, respectively to 3.1 ± 0.2, 6.1 ± 0.2 and 8.9 ± 0.1% d.m., and an increase in crude fiber respectively to 2.8 ± 0.3, 3.5 ± 0.3 and 3.9 ± 0.3% d.m. In the case of walnuts, the increase in the fat content in individual samples of the dough was even greater, and amounted to 4.0 ± 0.1, 7.2 ± 0.1 and 10.0 ± 0.1% d.m. However, the content of fiber in these samples was at a similar level, and amounted to 2.9 ± 0.1, 3.4 ± 0.2 and 3.5 ± 0.3% d.m. The content of fat and crude fiber was determined according to the ICC Standard [8] and the AOAC Standard, respectively [9].

### 2.2. Methods 

#### 2.2.1. Gluten Content

The wet gluten content in the analyzed systems was determined according to the Polish standard [10]. A weighed sample of flour (50 g) was transferred to a mortar, 25 mL tap water was gradually added, and a dough was made by kneading with a pestle. Then, the dough was formed into a ball, placed in a beaker, poured until completely covered with water and left for 20 min. The dough was then washed in a gentle stream of water by kneading with the fingers over the sieve. Gluten was washed away until tap water leaking through the sieve showed no reaction with Lugol’s solution for the presence of starch. After the elution was completed, the particles that fell on the sieve were attached to the mass of eluted gluten; the sample was then dried in a canvas and weighed. The final result is based on the weight of flour with a moisture content of 14%. The analysis was performed in duplicate.

#### 2.2.2. Farinographic and Extensographic Analysis

Changes in the consistency and elasticity of the dough during its formation and mixing were determined by means of a farinograph (Farinograph-E, Brabender, Duisburg, Germany), in accordance with the PN-ISO 5530-1:1999 standard [11]. As a part of this analysis, the water absorption of flour (%), dough development time (min), dough stability (min), degree of softening (FU) after 10 min, and farinographic number were determined. The analysis was performed in triplicate. In turn, the analysis of the dough extensibility was carried out using an extensograph (Extensograph-E, Brabender, Duisburg, Germany) in accordance with the PN-ISO 5530-2: 2004 standard [12]. As a part of this test, the energy of the dough (cm^2^), resistance of extension (BU), extensibility (mm), maximum (BU), and ratio number were determined. The analysis was performed in duplicate.

#### 2.2.3. Frequency Sweep Test

The viscoelastic properties of the tested dough were characterized at a temperature of 25 °C using a MARS II rheometer (Thermo Fisher Scientific, Waltham, MA, USA) equipped with a system of serrated parallel plates (diameter 35 mm, gap size 1 mm). The samples of the dough obtained by thoroughly mixing the ingredients with the optimal amount of water determined for the control sample in the farinographic analysis were placed in the measuring system of the rheometer and left for 3 min to relax the stresses and stabilize the temperature. In order to eliminate the drying of the dough during the analysis, silicone oil was used. The mechanical spectra were determined in the range of linear viscoelasticity at a constant strain amplitude of 0.1% in the angular frequency range of 1–100 rad/s. The experimental data were described by power Equations (1) and (2):

(1) $$G^{\prime }\left(\omega \right)=K\prime \cdot \omega^{n\prime }$$

(2) $$G\prime \prime \left(\omega \right)=K\prime \prime \cdot \omega^{n\prime \prime }$$

where: *G′* is the storage modulus (Pa), *G″* is the loss modulus (Pa), *ω* is the angular frequency (rad/s), and *K′*, *K″*, *n′* and *n″* are experimental constants [13]. 

The analysis was performed in triplicate.

#### 2.2.4. Creep and Recovery Test

The creep and recovery tests of the dough obtained as described above were carried out under the same conditions as the sweep frequency test, with a constant creep deformation of *τ*_0_ = 2 Pa for 120 s. The recovery phase lasted for 240 s. The experimental data were described using the Burgers model (Equations (3) and (4)):

(3) $$J\left(t\right)=J_{0}+\frac{t}{\eta_{0}}+J_{1}\cdot \left(1-exp^{-\frac{t}{\lambda_{ret}}}\right)\;\mathrm{for}\;\mathrm{the}\;\mathrm{creep}\;\mathrm{phase}$$

(4) $$J\left(t\right)=\frac{t_{1}}{\eta_{0}}-J_{1}\cdot \left(1-exp^{\frac{t_{1}}{\lambda_{ret}}}\right)\cdot exp^{-\frac{t}{\lambda_{ret}}}\;\mathrm{for}\;\mathrm{the}\;\mathrm{recovery}\;\mathrm{phase}$$

where: *J* is the compliance (Pa^−1^), *J*_0_ is the immediate compliance (Pa^−1^), *J*_1_ is the viscoelastic compliance (Pa^−1^), *η*_0_ is the zero shear viscosity (Pa∙s), *λ_ret_* is the retardation time (s), and *t*_1_ is the time after which the stress is removed (s).

#### 2.2.5. Analysis of the Texture Properties of the Dough

A dough texture analysis was performed along with the TPA test [14] using an EZ Test EZ-LX texturometer supported by the Trapezium X Texture Pl software (Shimadzu, Kyoto, Japan). The dough sample prepared in the farinograph was compressed twice to 50% deformation using a probe with a diameter of 25 mm. The squeezing of the dough sample was carried out at a speed of 50 mm min^−1^. The rest period between the cycles was 5 s. During the analysis, the following values were determined: hardness (N), elasticity (-), adhesiveness (J), cohesiveness (-) and chewiness (N). The determination was performed in five replications. 

#### 2.2.6. Statistical Analysis

The obtained results were subjected to a statistical analysis including a two-way ANOVA. In order to determine the significance of the differences between the mean values, Duncan’s test was performed at the significance level of *p* = 0.05. Additionally, between the parameters characterizing the properties of the dough, the values of Pearson’s linear correlation coefficients were calculated, and their significance was tested at the significance level of *p* = 0.05. The statistical analysis was performed using Statistica software, Version 13.3 (StatSoft, Krakow, Poland).

## 3. Results and Discussion

### 3.1. Farinograph Characteristics

**Figure 1:**
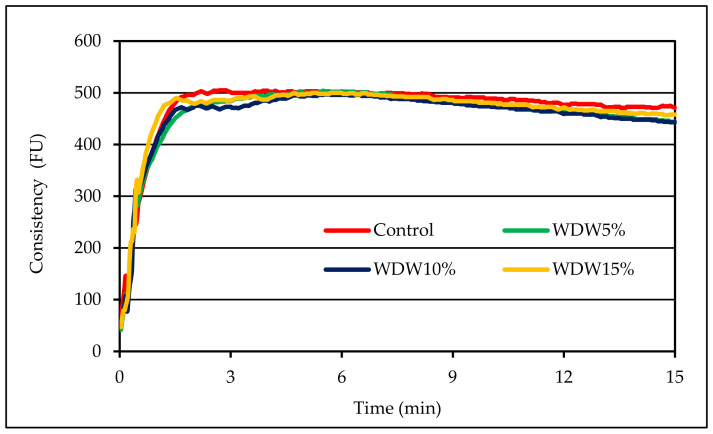
Curves from the farinographic analysis of the tested doughs.

The formation of the dough is an important step in the processing of flour and the production of further products from it. The proper formation of a continuous network of wheat gluten gives the dough both stickiness and elastic characteristics. It has been proven that the quality of wheat dough is directly regulated by the structure of the gluten network [15], and also by the interaction of other flour components such as non-gluten proteins, fiber and fat. This research showed that as a result of replacing a part of wheat flour with nut flour, the gluten content in the dough decreased (Table 1). The two-factor analysis of variance which we performed did not show a significant statistical effect of the type of nut flour or the interaction between the level of supplementation and the type of nut flour on the value of this parameter. The addition of hazelnut or walnut flour to the wheat flour resulted in a dough with different farinographic characteristics to the control sample. Figure 1 shows examples of charts from the farinographic analysis of the tested systems. 

Based on the analysis of these charts, it can be concluded that the addition of nut flour extended the development time of the dough. Doughs with the addition of nut flour were characterized by a higher degree of softening compared to the control sample. The two-factor analysis of variance showed a statistically significant effect of the type of nuts, the amount of this addition, and the interaction between these factors on the water absorption and development time of the dough (*p* < 0.001). It was found that the average water absorption of flour was lower in the dough enriched with flour with W in comparison to that with the addition of flour with H. The average value of this parameter for WDH was 48.6%, and in the case of WDW it was 47.9% (Table 1). The water absorption of the flour was strongly correlated with the content of wet gluten (r = 0.98, *p* < 0.05). Moreover, the value of this parameter decreased with the increase of the share of nuts in the dough. This is consistent with the observations of Gόmez et al. [6], who analyzed the effect of enrichment with nut paste (based on almonds, hazelnuts, walnuts and peanuts) on the rheological properties of wheat dough and bread. The cited authors showed that the water absorption of flour decreased with the increasing the share of nut paste in the system. The lowest water absorption was recorded for flour with the addition of hazelnut and walnut paste. The addition of almond and peanut paste resulted in the highest water absorption value.

According to the cited authors [6], changes in the water absorption of flour result from the presence of a large amount of fat and fiber in the tested systems. However, the replacement of part of the flour with nut paste was associated with a decrease in the proportion of the gluten proteins responsible for the structure of the dough in favor of non-gluten proteins. Non-gluten proteins show a high water absorption, and also compete for water with other food ingredients. Therefore, their high level may contribute to the increasing water absorption of the system. The presence of fiber also significantly affects the water absorption of flour. This is because, in the structure of the substances that are a part of the fiber, there is a very large number of hydroxyl groups, which easily—due to the formation of hydrogen bonds—interact with water molecules [6,15,16]. According to Liu et al. [15], fiber has a stronger ability to absorb water compared to other flour ingredients such as starch or proteins. However, as reported by Wanga et al. [16], the presence of fiber in the dough may affect both the deterioration and improvement of rheological characteristics, because the direction of the changes depends on the type, structure, particle size and amount of the added fiber. Numerous studies on the effects of buckwheat bran additives to wheat flour [17], barley bran [18], and grape skins [14] confirm this effect. Liu et al. [15] showed that with the increase in the proportion of fiber in the dough, the development time and stability of the dough increased, which may be due to the weakening of the gluten network due to fiber. In such systems, gluten must compete with fiber for water, which hinders the formation of a three-dimensional network. Nuts are a rich, natural source of fat; therefore, their addition to flour increases the proportion of this component in the system, and thus affects the water absorption of the flour and other farinographic features, such as the dough development time and stability.

The dough development time (DDT) is the time from adding water to the flour until the dough has the greatest resistance to mixing. During this phase, the water moistens the flour ingredients and the dough begins to develop [19]. It was found that the dough development time depended only on the type of nut flour (*p* < 0.001), and it was the shortest for the control sample (3.0 min). The use of nut flour increased the dough development time, but the added quantity did not significantly change the described parameter. WDW10% dough developed for the longest time. Gómez et al. [6] obtained similar results. The cited authors showed that the addition of nut paste increased the development time of the wheat dough as the proportion of this additive increased. However, they found no effect of the type of nut paste on the value of the DDT. The DDT determined using the consistensograph for the control sample was 124.00 s; the values for doughs with 5%, 10% and 15% addition of peanut paste were on average 146.38 s, 170.75 s and 211.50 s, respectively. This research showed that the control dough was the most stable, and the addition of ground nuts resulted in a statistically significant reduction in the value of this parameter. It was shown that both the DDT and stability significantly influenced the values of the dough firmness (*p* < 0.001) and the softening. The average value of the dough firmness for the WDH dough was 7.4 min, and for the WDW dough was 9.7 min. The softening, instead, was the lowest for the control sample, and amounted to 21.3 BU. The addition of nut flour significantly increased the softening of the dough; the more was added, the softer the dough was. For example, for WDH, the softening ranged from 27.3 FU (WDH 5%) to 53.0 FU (WDH 15%). As a result of the statistical analysis, significant linear correlations were found between the stability of the dough and the degree of softening, as well as the farinographic number (r = −0.95, r = 0.99, *p* < 0.05, respectively).

### 3.2. Extensograph Characteristics 

Extensograph analysis gives information on the viscoelastic behavior of the dough. The extensograph measures the extensibility of the dough, and its resistance to extension. The combination of good toughness and good extensibility results in the desired dough properties [2]. In addition, the results of extensographic analysis allow the assessment of the impact of the fermentation process of the dough—interrupted at various times by the piercing process—on its rheological properties, and especially on the tensile strength [20]. The parameters of the extensographic analysis were determined after 30, 60 and 90 min of fermentation. The energy of the dough is a parameter that describes the quality of the flour used, as well as the influence of the technological additives. The two-factor analysis of variance showed a significant statistical effect of the type of nut flour and the interaction of both tested factors on the value of the energy of the dough (*p* < 0.001). This parameter describes the energy input required to stretch the dough. The energy of the control sample after 30 min of fermentation was shown to be 100.5 cm^2^. The average energy of WDH after 30, 60 and 90 min of fermentation was lower than that of the control and WDW (Table 2 and Table 3). As the fermentation time passed, the energy of all of the tested dough types generally increased. According to Boyacioğlu i D’Appolonia [21], flour an energy value in the range of 120 cm^2^ to 200 cm^2^ is considered to be strong. The resistance to extension of the dough increased as the fermentation process progressed. Statistical analysis showed that the type of nut flour did not significantly affect the value of this parameter. The average tensile resistance of the dough increased with the addition of nut flour, and was higher by about 10 BU in comparison with the control sample (Table 2). Nevertheless, as the proportion of nuts in the dough increased, the resistance to extension decreased. The extensibility of the dough is a parameter that allows us to determine the length of the fermentation process. The analysis of variance showed that the type of nut flour and the amount of its addition to the dough had a statistically significant effect on the value of this parameter (*p* < 0.001). The control sample had an elongation of 177.0 mm, while the addition of hazelnut or walnut flour reduced or increased the extensibility of the dough, respectively. The average values of extensibility after 30 min of WDH and WDW were 167 mm and 189 mm, respectively, i.e., it was respectively lower and higher by about 6% and 7% compared to the control sample. Moreover, as the fermentation process progressed, the extensibility of all of the analyzed doughs increased. Gómez et al. [6], by conducting an alveograph analysis of wheat dough enriched with nut paste, showed that the extensibility of the dough increased as the proportion of nut paste in the dough increased. However, they noticed significant differences only between the 5% and 15% additions of nut paste. No differences between the control and the enriched dough were found. According to Boyacioğlu and D’Appolonia [21], the minimum extensibility of the dough should equal 120 mm; the higher it is, the better the flour quality. Thus, the analyzed doughs fall within this range. 

The performed statistical analysis showed a number of significant linear correlations between the extensographic parameters characterizing the dough after different fermentation times. Thus, the energy of the dough after fermentation at 30 min correlated with the extensibility of the dough (30 min) (r = 0.84, *p* < 0.05) and the parameters determined after a 60-min fermentation, i.e., with the dough energy and extensibility (r = 0.87, r = 0.90, *p* < 0.01), and with the dough energy after 90 min of fermentation (r = 0.79, *p* < 0.05).

### 3.3. Sweep Frequency Test

**Figure 2:**
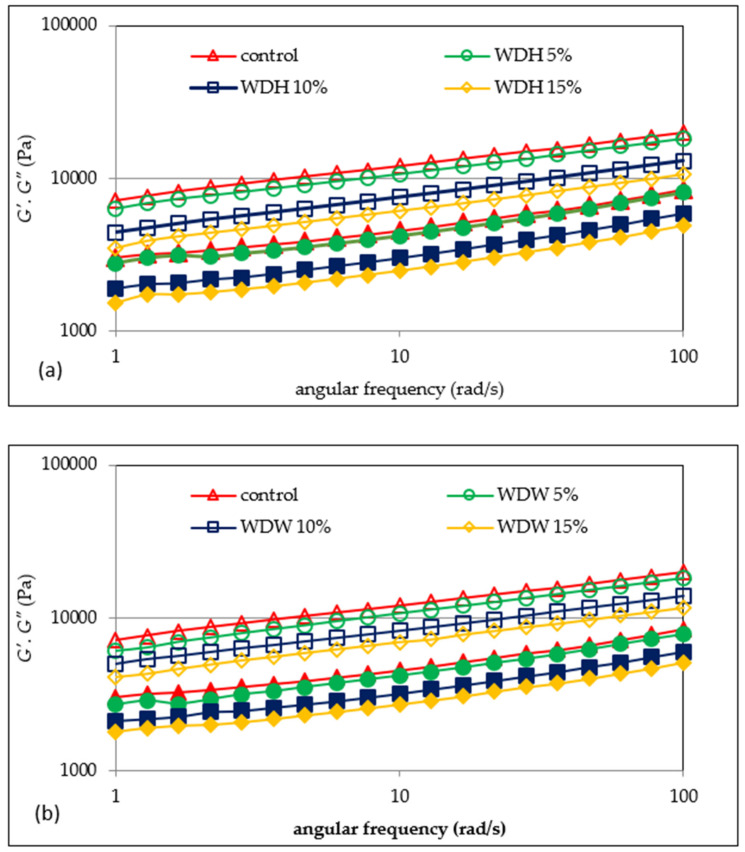
Mechanical spectra of doughs with the addition of hazelnut (**a**) or walnut (**b**) flour. *G′* denotes an empty marker; *G″* denotes a filled marker.

**Figure 3:**
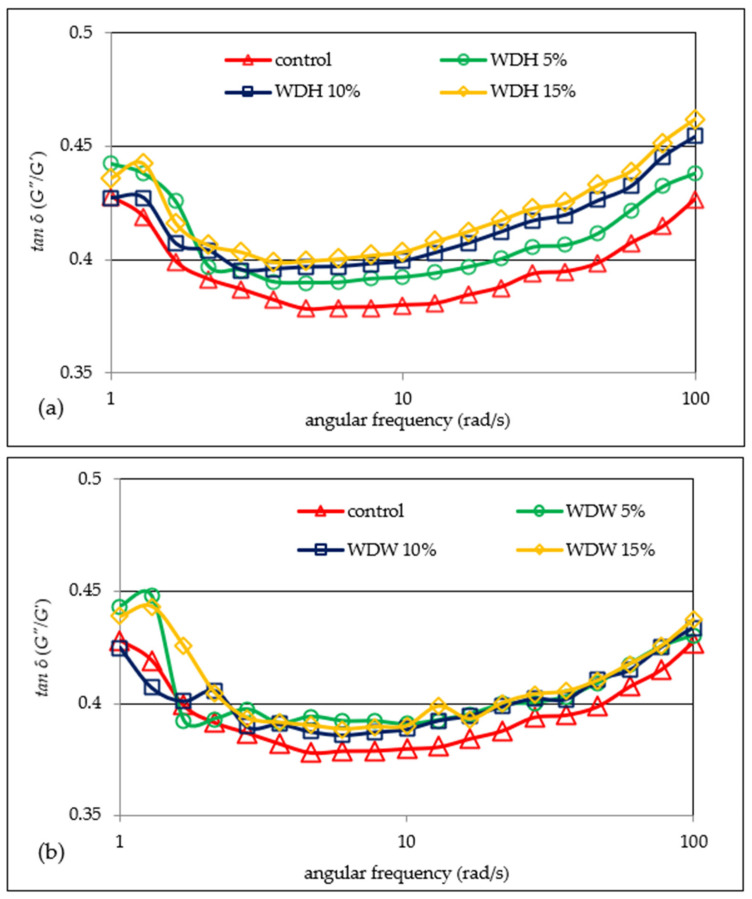
Tangent δ’s dependence on the angular frequency of the analyzed doughs with the addition of hazelnut (**a**) or walnut (**b**) flours.

The rheological analysis is related to the sample structure, and is based on the examination of the horizontal and vertical deformations of the matter. Dynamic rheology is analyzed by means of an oscillating rheometer, and it enables the analysis of viscoelastic properties [15]. The parameters describing the share of viscous and elastic features in the material are the storage modulus (*G′*) and the loss modulus (*G″*). The storage modulus (*G′*) describes the share of elastic features, and corresponds to the part of the energy that is stored. The loss modulus (*G″*) corresponds to the viscous features, and reports the part of the energy which is lost during sinusoidal deformation [4,22]. Figure 2a,b show the mechanical spectra of dough with the addition of hazelnuts and walnuts, and Figure 3a,b show changes in the tangent value of the phase shift angle with respect to the angular frequency for the tested dough.

On the basis of the obtained test results, it was found that the values of the *G′* and *G″* modules increased with the increasing frequency. Thus, recovering energy from a stressed system is a slow process, as the system is not completely elastic [3,15]. Moreover, the values of the storage modulus (*G′*) were greater than those of the loss modulus (*G″*). Thus, in all of the analyzed doughs, elastic features prevailed over sticky features. It is worthy of note that the values of the *G′* and *G″* modules decreased with the increasing share of both hazelnut flour (Figure 2a) and walnut flour (Figure 2b) in the dough. This tendency is probably attributable to the increased fat content in the dough, which resulted in a looser consistency, and the reduction in gluten content in the dough as a result of the replacement of part of the wheat flour with nut flour. 

A similar trend has been shown in previous studies [22] analyzing the viscoelastic properties of gruels based on wheat flour systems with hazelnuts or walnuts. In the presence of water, gluten forms a three-dimensional net that provides the springy nature of the dough. Meanwhile, Liu et al. [15] noticed an increase in the value of the *G′* and *G″* modules as the addition of wheat bran fiber to the dough increased. Similar conclusions were drawn by Mironeasa et al. [14], who analyzed the rheological properties of wheat dough with the addition of grape skin flour (GPF), which is rich in fiber. According to the cited authors [14,15], this is due to a reduction of the dough’s hydration as a result of a competition between fiber and gluten for water, or due to fiber filling the viscoelastic structure. In all probability, a similar situation could be observed in the case of the analyzed WDH and WDW doughs. Moreover, this phenomenon is exacerbated as the proportion of fiber in the dough increases. Mironeasa et al. [14] found that the *G′* and *G″* modulus values increased as the particle size of the added GPF decreased, but only to a level of 7%. The cited authors explained this fact by the increased water absorption capacity of smaller GPF particles. The reduction in the values of the *G′* and *G″* modules with an addition of GPF of more than 7% may result from the weakening and disturbance of the continuous gluten network during dough formation due to the reduced level of gluten in the system due to the replacement of part of the flour with GPF. This is confirmed by the presented research results. Meanwhile, Peressini et al. [23] found that the presence of soluble fiber may increase the flexibility and strength of the dough. In addition to fiber, another factor that may affect the viscoelastic properties of the dough is fat. Agyare et al. [3] found a decrease in the values of the *G′* and *G″* modules due to the addition of shortening to wheat dough, and this tendency increased as the degree of substitution increased. According to Watanabe et al. [24] and Agyare et al. [3], the added fat seems to uniformly distribute the gluten gel between the starch granules in the dough, reducing friction between the starch granules, thus resulting in a lower *G′*. Therefore, this is a confirmation of the results presented in this study. Because nuts contain an average of 50–70% fat [25], their addition contributes to the increase of this ingredient in the dough. Agyare et al. [3] indicated that when fat is added to a dough, the rheological properties are also influenced by the consistency of the fat. This is because the dough containing shortening lipids with a liquid consistency was characterized by a lower value of the *G′* and *G″* modules compared to the dough with the addition of shortening lipids with a solid consistency. The ratio of the energy lost to the energy stored in each cycle is described as *tan δ,* which indicates the physical behavior of the system [22]. It was found that the values of the tangent of the phase shift angles (*tan δ* = *G″/G′*) for both the hazelnut and walnut doughs initially decreased, and then increased as the frequency increased (Figure 3a,b). This may indicate the formation of the structure of the dough [15]. The values of *tan δ* indicate that the tested doughs have the character of weak structures, while the presence of nuts in the system intensified this tendency, which is stronger the greater the share of nuts in the system. Earlier studies by Pycia and Juszczak [22] also showed that the gels of the wheat flour–nut systems were weak gels.

The values of the parameters of the power equations describing the mechanical spectra of the tested WD with the addition of flour based on H and W were presented in Table 4. The two-factor analysis of variance showed a significant statistical effect of the type of nut flour and the interaction between the type of nut flour and the amount of its addition to wheat flour (*p* < 0.001) on the value of the parameter *K′,* which represents the initial value of the module *G′*. It was shown that the highest value of this parameter was found in the control wheat dough, and the addition of nut flour resulted in a significant reduction in this value. Thus, the mean *K′* values of the WDH and WDW samples were 4829.9 and 5100.0 Pa·s*^n′^*, respectively, and they were lower by approximately 33% and 29%, respectively, compared to the control. Thus, replacing part of WF with walnut or hazelnut flour resulted in a greater reduction in the value of the *K′* parameter compared to the value of WDW. The *K″* factor indicates the initial value of the *G″* modulus. In the case of this parameter, the two-factor analysis of variance also showed a significant statistical effect of the type of nut flour, the amount of its addition, and the interaction between the type of nut flour and the amount of its addition to the dough (*p* < 0.001) on its value. In this case, too, a decrease in the value of this parameter was observed with the addition of nut flour to the dough. In the case of WDH dough, the average value of the parameter was 1948.6 Pa·s^n″^, and it was lower by 38% than the control sample. The mean value of the *K″* parameter for WDW was 28% lower than that for the control sample. In the case of the values of the parameters *n′* and *n″*, indicating the sensitivity of the modules to changes in angular velocity, no influence of the tested factors on the value of these parameters was found. According to Song and Zheng [4], the analysis of the viscoelastic properties of the dough does not allow us to fully predict the baking value of wheat flour; therefore, other methods using a farinograph, extensograph, or a trial baking in the laboratory are helpful. The performed statistical analysis confirmed the existence of a strong linear correlation between the parameters of sweep frequencies *K′* and *K″* and the water absorption of flour (r = 0.91, r = 0.89, and *p* < 0.05, respectively).

### 3.4. Creep and Recovery Test

**Figure 4:**
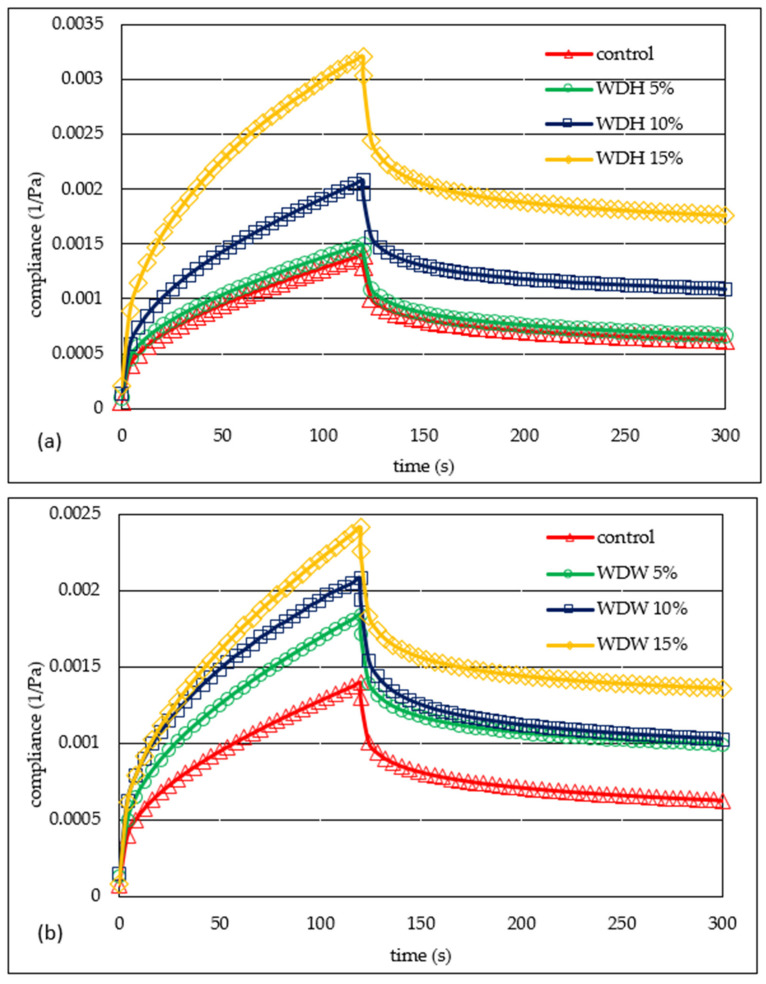
Creep and recovery curves of the control sample and doughs with hazelnut (**a**) or walnut (**b**) flours.

The creep–recovery curves of the doughs showed a typical viscoelastic behavior of the system combining both viscous fluid and elastic characteristics. The strain of the dough system changed continuously with time [15]. Figure 4a,b show the exemplary creep and recovery curves of the studied systems. All of the curves have a shape which is characteristic of viscoelastic materials. The deformation of the dough continuously changed over time. It was shown that the control sample was characterized by the lowest susceptibility to deformation, while the addition of nuts increased the susceptibility. This shows a decrease in resistance to the applied stress. In turn, the increase in the resistance of the dough to deformation under the influence of the addition of dietary fiber from wheat bran was observed by Liu et al. [15]. According to the cited authors, the dough with this addition was stiffer than the control sample. This phenomenon is explained by the increased water absorption of fiber, which reduces the hydration of starch and protein. Mironeas et al. [14] found that the addition of grape skins with small particle sizes to wheat dough in an amount below 5% increases the susceptibility of the dough to applied stress. On the other hand, the addition of this component in an amount exceeding 5% caused an increase in the value of the *G′* and *G″* modules. On the other hand, the presence of medium and large particles of grape skins in the dough caused a decrease in the resistance of the dough to deformation. This was due to the high water absorption of fiber. Thus, the adequate hydration of the gluten allows the free formation of the gluten network and thus the formation of a flexible dough. Problems with starch and protein hydration resulting from the presence of a large amount of fat or fiber lead to the production of a stiff, deformation-resistant dough. In the analyzed case, as a result of the replacement of some of the wheat flour with flour based on nuts, their amount in the individual doughs also decreased. This is resulted in the production of a dough which was more susceptible to deformation. According to Hüttner et al. [26], the flexibility of a dough, i.e., its susceptibility to deformation over time, is significantly related to its hydration capacity, which results from the size of the flour particles; the degree of starch damage; the level of protein, including gluten; and the presence of non-starch substances and substances of a hydrophobic nature.

Table 5 presents the values of the parameters of the Burgers model used to describe the susceptibility to deformation of the tested dough. The applied model describes well the experimental data (R^2^ > 0.9752). The two-factor analysis of variance showed a significant statistical effect of the amount of the addition of nut flour, and the interaction between the type of nut flour and the amount of its addition (*p* < 0.001) on the value of the immediate and viscoelastic compliance parameters. The viscoelasticity values also significantly depend on the type of nut flour added to the dough. Immediate compliance (*J*_0_) is related to the tensile energy of elastic bonds, which disappears immediately after the force dissipates [22]. The lowest value of immediate compliance was found for the control sample, while the addition of nuts increased the value of this parameter. The mean values of *J*_0_ for WDH and WDW compared to the control were higher by 33% and 19%, respectively. An inverse relationship was demonstrated by Pycia and Juszczak [22] when analyzing the value of immediate compliance for gels of wheat flour supplemented with hazelnuts or walnuts. This may probably be due to the type of material being tested. In this work, the dough was tested, while in the previous work used the gels, i.e., systems after thermal treatment containing a much larger amount of water. Viscoelastic compliance (*J*_1_) is related to the destruction or transformation of bonds in the material [22]. The control sample had the lowest value of this parameter, and the highest WFW at 15%. The presence of HF in the dough resulted in an increase in its viscoelasticity compared to the flour based on W (Table 5). It was shown that all of the analyzed factors had a statistically significant (*p* < 0.001) effect on the value of the viscosity parameter at zero shear (*η*_0_). The control sample had the highest value of this parameter. Therefore, the addition of nuts decreased its value. Doughs with walnuts (WDW) were characterized by a value of *η*_0_ lower by 11,000 Pa·s compared to the WDH. The retardation time characterizes the time necessary for the viscoelastic material to respond to the applied stress [22]. The highest value of this parameter was found in the control sample (18.6 s), and the lowest value was for WDH at 15%. The average value of *λ_ret_* for doughs with the addition of HF and WF was lower than the control sample by 4.4 s and 5.5 s, respectively. The performed statistical analysis confirmed the existence of a strong linear correlation between the parameters *η*_0_ and *λ_ret_* and the water absorption of flour (r = 0.88, r = 0.86, *p* < 0.05, respectively). Moreover, the *J*_0_ parameter was strongly correlated with the parameters of the farinographic analysis, such as the stability, degree of softening and farinographic number (respectively: r = −0.76, r = 0.85; r = −0.78, *p* < 0.05), as well as with the parameters *K′, K″, J*_1_*,* and *λ_ret_* (respectively: r = −0.78, r = −0.79; r = 0.96, r = −0.79, *p* < 0.05).

### 3.5. Textural Properties 

Dough is an intermediate product between flour and bakery products, and its rheological properties are of great importance in bread production, because they affect the dough processing and the quality of the final product [14]. In addition to basic rheological methods, including the analysis of low-strain viscoelastic properties, high-strain texture measurements can be used to assess the rheology of the dough. In this study, the analysis of the texture profile was used, the results of which are summarized in Table 6. The statistical analysis performed showed that only in the case of dough hardness was the value of the parameter significantly influenced by the degree of supplementation of wheat flour with nut flour. In the case of the remaining parameters, none of the tested factors statistically significantly affected their value.

The control dough was characterized by the highest hardness, while the addition of nut flour significantly decreased the value of this parameter. The average values of the hardness of the WDH and WDW doughs were 5.40 N and 5.59 N, respectively, which were lower than the control sample by 22% and 19%, respectively. A clear trend was also observed in the changes of elasticity, the values of which increased with the increase in the share of nut flour, but these changes were not statistically significant. There was no significant statistical effect of any of the tested factors on the values of the adhesiveness, cohesiveness or chewiness of the tested doughs. In the case of chewiness, the highest value of this parameter was found for the WDH 15% dough. Such a high value of chewiness for this sample resulted from its greatest elasticity and cohesiveness. The statistical analysis confirmed the existence of a strong linear correlation between the hardness of the dough and the water absorption of flour, the content of wet gluten (r = 0.89, r = 0.90, *p* < 0.05, respectively) and the parameters *K′* and *K″* (r = 0.90, r = 0.91, *p* < 0.05, respectively), as well as with the parameters *J*_0_ and *η_0_* (r = −0.81, r = 0.82, *p* < 0.05, respectively). Moreover, the dough hardness was significantly correlated with the dough cohesiveness (r = −0.83, *p* < 0.05). The dough elasticity statistically significantly correlated with parameters such as the water absorption of the flour (r = −0.78, *p* < 0.05), the wet gluten content (r = −0.80, *p* < 0.05), and parameters *J*_1_*, η_0_,* and *λ_ret_* (r = 0.82, r = 0.80, r = −0.77; *p* < 0.05).

## 4. Conclusions

The studies assessed the effect of the addition of walnuts or hazelnuts on the rheological properties of wheat dough. It was found that the ground nuts can weaken the structure of the dough and deteriorate its rheological properties. It was shown that their presence lowers the water absorption of the flour, lengthens the development time of the dough, reduces its stability during kneading, and increases its degree of softening. This was confirmed by the results of analyzes of viscoelastic properties in the field of small deformations. The doughs with the addition of nut flour were characterized by greater flexibility, i.e., they were more susceptible to deformation compared to the control sample. This effect was more observable in the case of the dough with walnut flour than in the dough with hazelnut flour, and with the increase in the share of nut flour in the recipe. This is probably due to the differences in the fat content of the two nut flours. Thus, the study showed a not-very-favorable effect of the addition of walnut flour on the structure of wheat dough, which may be a direct result of the effect of fat, fiber and walnut proteins on the difficulty in forming the gluten structure. It should also be taken into account that by replacing some of the flour with ground nuts, the mixture was deprived of a certain dose of starch and gluten, which are directly responsible for the structure of the dough. Nevertheless, in the literature there is evidence of a beneficial effect of nuts on the nutritional value, health benefits and sensory characteristics of bread, such that the right proportion of nut flour and wheat flour should be chosen.

## Figures and Tables

**Table 1:** Wet gluten content and farinographic properties of the tested doughs.

Sample	Wet Gluten Content * (%)	Water Absorption ** (%)	Dough Development Time ** (min)	Dough Stability ** (min)	Degree of Softening ** (FU)	Farinograph Number **
Control	27.5 ^d^ ± 0.1	54.9 ^e^ ± 0.7	3.0 ^a^ ± 1.1	11.6 ^d^ ± 1.1	21.3 ^a^ ± 6.1	122.7 ^c^ ± 10.2
WDH5%	25.4 ^c^ ± 0.2	50.6 ^d^ ± 0.1	5.4 ^b^ ± 0.5	9.4 ^b^ ± 0.2	27.3 ^b^ ± 2.1	103.3 ^b^ ± 4.0
WDH10%	23.8 ^b^ ± 0.4	48.6 ^c^ ± 0.2	4.7 ^bc^ ± 0.7	6.8 ^a^ ± 0.2	47.7 ^c^ ± 4.7	78.3 ^a^ ± 6.0
WDH15%	22.4 ^a^ ± 0.2	46.8 ^b^ ± 0.2	4.3 ^b^ ± 0.4	5.9 ^a^ ± 0.1	58.0 ^c^ ± 2.6	72.7 ^a^ ± 2.5
WDW5%	25.1 ^c^ ± 0.3	50.4 ^d^ ± 0.6	5.4 ^b^ ± 0.1	9.0 ^b^ ± 0.1	28.0 ^b^ ± 3.0	103.0 ^b^ ± 4.4
WDW10%	23.4 ^b^ ± 0.8	48.5 ^c^ ± 0.3	5.6 ^b^ ± 0.3	9.6 ^bc^ ± 0.6	29.7 ^b^ ± 4.9	102.3 ^b^ ± 7.8
WDW15%	22.2 ^a^ ± 0.2	45.0 ^a^ ± 0.2	5.2 ^bc^ ± 0.7	10.5 ^c^ ± 1.1	26.7 ^b^ ± 6.2	108.3 ^b^ ± 8.5
	two-factor ANOVA is the *p*					
Factor 1	*p* = 0.735	*p* < 0.001	*p* < 0.001	*p* < 0.001	*p* < 0.001	*p* < 0.001
Factor 2	*p* < 0.001	*p* < 0.001	*p* = 0.159	*p* < 0.001	*p* < 0.001	*p* < 0.001
F1 × F2	*p* = 0.736	*p* < 0.001	*p* = 0.243	*p* < 0.001	*p* < 0.001	*p* < 0.001

* Mean values from three repetitions ± SD; ** mean values from three repetitions ± SD. Values in columns followed by the same superscript letters do not significantly differ at the significance level of 0.05. Factor 1 is the type of nut flour; Factor 2 is the supplementation level.

**Table 2:** Extensographic properties of the tested wheat doughs with the addition of nut flour.

Sample	Energy (cm^2^)			Resistance to Extension (BU)			Extensibillity (mm)		
	30 min	60 min	90 min	30 min	60 min	90 min	30 min	60 min	90 min
Control	100.5 ^b^ ± 3.5	102.0 ^ab^ ± 9.9	102.0 ^ab^ ± 9.9	299.0 ^ab^ ± 9.9	338.5 ^a^ ± 9.2	403.5 ^ab^ ± 12.0	177.0 ^bc^ ± 5.7	169.5 ^b^ ± 9.0	148.0 ^b^ ± 1.4
WDH5%	89.0 ^a^ ± 0.0	97.0 ^a^ ± 4.2	97.0 ^a^ ± 4.2	347.5 ^c^ ± 3.5	417.0 ^c^ ± 19.8	432.0 ^b^ ± 29.7	153.0 ^a^ ± 1.4	141.0 ^a^ ± 4.2	139.0 ^a^ ± 5.7
WDH10%	90.0 ^a^ ± 0.0	94.0 ^a^ ± 12.7	94.0 ^a^ ± 12.7	298.0 ^ab^ ± 12.7	348.5 ^ab^ ± 4.9	354.0 ^a^ ± 18.4	170.0 ^b^ ± 5.7	162.5 ^b^ ± 2.1	156.0 ^ab^ ± 14.1
WDH15%	90.0 ^a^ ± 1.4	98.0 ^ab^ ± 1.4	98.0 ^ab^ ± 1.4	287.5 ^a^ ± 0.7	334.0 ^a^ ± 17.0	373.5 ^a^ ± 36.1	178.5 ^bc^ ± 0.7	162.5 ^b^ ± 4.9	168.0 ^bc^ ± 9.9
WDW5%	109.0 ^c^ ± 1.5	127.0 ^c^ ± 5.7	127.0 ^c^ ± 5.7	316.5 ^b^ ± 12.0	352.5 ^ab^ ± 10.6	396.0 ^ab^ ± 1.4	180.0 ^cd^ ± 1.4	176.5 ^bc^ ± 2.1	181.0 ^c^ ± 7.1
WDW10%	108.0 ^c^ ± 1.4	107.0 ^ab^ ± 5.7	107.0 ^ab^ ± 5.7	302.5 ^ab^ ± 4.9	351.0 ^ab^ ± 14.1	359.5 ^a^ ± 2.1	188.0 ^d^ ± 4.2	177.5 ^bc^ ± 9.2	166.5 ^bc^ ± 10.6
WDW15%	119.0 ^c^ ± 4.2	114.0 ^bc^ ± 2.8	114.0 ^bc^ ± 2.8	307.0 ^ab^ ± 9.9	376.5 ^b^ ± 6.4	369.0 ^a^ ± 14.1	200.0 ^e^ ± 4.2	192.0 ^c^ ± 12.7	174.0 ^bc^ ± 7.1
two-factor ANOVA is the *p*									
Factor 1	*p* < 0.001	*p* < 0.001	*p* < 0.001	*p =* 0.654	*p =* 0.042	*p =* 0.379	*p* < 0.001	*p* < 0.001	*p* < 0.001
Factor 2	*p =* 0.014	*p* < 0.001	*p =* 0.117	*p* < 0.001	*p* < 0.001	*p =* 0.021	*p* < 0.001	*p* < 0.001	*p =* 0.276
F1 × F2	*p* < 0.001	*p* < 0.001	*p =* 0.223	*p* < 0.001	*p* < 0.001	*p =* 0.411	*p =* 0.259	*p =* 0.169	*p =* 0.070

Mean values from two repetitions ± SD. Values in columns followed by the same superscript letters do not significantly differ at a significance level of 0.05. Factor 1 is the type of nut flour; Factor 2 is the supplementation level.

**Table 3:** Extensographic properties of the tested wheat doughs with the addition of nut flour.

Sample	Maximum (BU)			Ratio Number		
	30 min	60 min	90 min	30 min	60 min	90 min
Control	418.0 ^bc^ *±* 9.9	466.0 ^b^ *±* 18.3	523.5 ^b^ *±* 41.7	1.7 ^ab^ *±* 0.1	2.0 ^a^ *±* 0.1	2.8 ^b^ *±* 0.1
WDH5%	419.5 ^bc^ *±* 10.6	498.0 ^bc^ *±* 14.1	515.0 ^b^ *±* 32.5	2.2 ^c^ *±* 0.1	2.9 ^b^ *±* 0.2	3.2 ^c^ *±* 0.4
WDH10%	383.0 ^a^ *±* 9.9	457.0 ^ab^ *±* 16.9	414.0 ^a^ *±* 33.9	1.8 ^b^ *±* 0.1	2.1 ^a^ *±* 0.1	2.3 ^a^ *±* 0.1
WDH15%	361.0 ^a^ *±* 5.7	413.5 ^a^ *±* 10.7	435.0 ^a^ *±* 12.7	1.6 ^ab^ *±* 0.0	2.1 ^a^ *±* 0.0	1.9 ^a^ *±* 0.5
WDW5%	443.5 ^c^ *±* 12.0	491.0 ^bc^ *±* 11.3	540.0 ^c^ *±* 4.2	1.8 ^ab^ *±* 0.1	1.9 ^a^ *±* 0.1	2.2 ^a^ *±* 0.1
WDW10%	410.0 ^b^ *±* 12.7	473.0 ^b^ *±* 43.8	494.5 ^b^ *±* 7.8	1.7 ^ab^ *±* 0.1	2.0 ^a^ *±* 0.1	2.2 ^a^ *±* 0.2
WDW15%	434.5 ^bc^ *±* 10.6	529.5 ^c^ *±* 3.5	493.5 ^b^ *±* 4.9	1.6 ^a^ *±* 0.1	2.0 ^a^ *±* 0.1	2.1 ^a^ *±* 0.0
two-factor ANOVA is the *p*						
Factor 1	*p* < 0.001	*p* < 0.001	*p* < 0.001	*p* < 0.001	*p* = 0.328	*p* = 0.110
Factor 2	*p* < 0.001	*p* = 0.185	*p* < 0.001	*p* < 0.001	*p* = 0.121	*p* = 0.024
F1 × F2	*p* < 0.001	*p* < 0.001	*p* = 0.495	*p* = 0.058	*p* = 0.351	*p* = 0.031

Mean values from two repetitions ± SD. Values in columns followed by the same superscript letters do not significantly differ at a significance level of 0.05. Factor 1 is the type of nut flour, Factor 2 is the supplementation level.

**Table 4:** Parameters of the power law equations describing the viscoelastic properties (25 °C) of wheat dough with nut flour.

Sample	*K′*	*n′*	R^2^	*K″*	*n″*	R^2^
Control	7264.9 ^f^ ± 30.1	0.22 ^a^ ± 0.01	0.9987	2848.6 ^g^ ± 71.0	0.22 ^a^ ± 0.01	0.9851
WDH5%	6419.1 ^g^ ± 137.7	0.23 ^ab^ ± 0.00	0.9984	2602.6 ^f^ ± 122.9	0.23 ^a^ ± 0.01	0.9748
WDH10%	4453.2 ^c^ ± 101.0	0.23 ^ab^ ± 0.01	0.9994	1775.8 ^c^ ± 66.6	0.25 ^a^ ± 0.01	0.9872
WDH15%	3617.4 ^a^ ± 45.9	0.23 ^ab^ ± 0.01	0.9973	1467.3 ^a^ ± 16.7	0.25 ^a^ ± 0.01	0.9838
WDW5%	6165.5 ^e^ ± 175.0	0.24 ^b^ ± 0.01	0.9980	2490.7 ^e^ ± 101.2	0.24 ^a^ ± 0.01	0.9876
WDW10%	5001.0 ^d^ ± 211.3	0.22 ^a^ ± 0.01	0.9988	1970.9 ^d^ ± 96.4	0.23 ^a^ ± 0.01	0.9852
WDW15%	4134.9 ^b^ ± 40.3	0.22 ^a^ ± 0.01	0.9986	1684.8 ^b^ ± 52.5	0.22 ^a^ ± 0.02	0.9835
	two-factor ANOVA is the *p*					
Factor 1	*p* < 0.001	*p =* 0.357		*p* < 0.001	*p =* 0.077	
Factor 2	*p* < 0.001	*p =* 0.416		*p* < 0.001	*p =* 0.085	
F1 × F2	*p* < 0.001	*p =* 0.093		*p* < 0.001	*p =* 0.078	

Mean values from three repetitions ± SD. *K′*, *K″*, *n′* and *n″* are the power law equations’ constants. Values in columns followed by the same superscript letters do not significantly differ at a significance level of 0.05. Factor 1 is the type of nut flour; Factor 2 is the supplementation level.

**Table 5:** Values of the Burgers model parameters for the creep and recovery curves of the wheat doughs with nut flour.

Sample	*J*_0_ (× 10^−4^) (Pa^−1^)	*J*_1_ (× 10^−4^) (Pa^−1^)	*η*_0_ (× 10^4^) (Pa·s)	*λ_ret_* (s)	R^2^
Control	2.0 ^a^ ± 0.1	5.2 ^a^ ± 0.7	17.7 ^f^ ± 0.8	18.6 ^c^ ± 1.1	0.9787
WDH5%	2.1 ^a^ ± 0.2	5.7 ^b^ ± 0.7	16.5 ^e^ ± 0.9	17.0 ^b^ ± 1.9	0.9752
WDH10%	2.6 ^ab^ ± 0.1	7.1 ^c^ ± 0.6	10.4 ^c^ ± 0.4	13.5 ^a^ ± 0.6	0.9835
WDH15%	3.8 ^c^ ± 0.6	11.0 ^d^ ± 0.3	6.5 ^a^ ± 0.1	11.9 ^a^ ± 0.7	0.9830
WDW5%	2.3 ^ab^ ± 0.1	6.0 ^b^ ± 0.4	11.5 ^d^ ± 0.5	13.4 ^a^ ± 0.5	0.9834
WDW10%	2.8 ^b^ ± 0.4	7.6 ^c^ ± 0.7	10.9 ^cd^ ± 0.5	13.6 ^a^ ± 0.8	0.9820
WDW15%	2.4 ^ab^ ± 0.3	7.8 ^c^ ± 0.6	8.5 ^b^ ± 0.3	12.2 ^a^ ± 1.0	0.9856
	two-factor ANOVA is the *p*				
Factor 1	*p =* 0.071	*p* < 0.001	*p* < 0.001	*p =* 0.047	
Factor 2	*p* < 0.001	*p* < 0.001	*p* < 0.001	*p* < 0.001	
F1 × F2	*p* < 0.001	*p* < 0.001	*p* < 0.001	*p* < 0.001	

Mean values from three repetitions ± SD. Values in columns followed by the same superscript letters do not significantly differ at a significance level of 0.05. Factor 1 is the type of nut flour, Factor 2 is the supplementation level.

**Table 6:** Textural properties of the tested doughs.

Sample	Hardness (N)	Elasticity (-)	Adhesiveness (J)	Cohesiveness (-)	Chewiness (N)
Control	6.92 ^e^ ± 0.19	1.27 ^a^ ± 0.07	−0.05 ^a^ ± 0.02	0.86 ^ab^ ± 0.16	7.66 ^b^ ± 1.54
WDH5%	6.31 ^cd^ ± 0.43	1.37 ^a^ ± 0.66	−0.05 ^a^ ± 0.02	0.83 ^a^ ± 0.09	4.95 ^a^ ± 0.17
WDH10%	5.53 ^b^ ± 0.66	1.34 ^a^ ± 0.41	−0.06 ^a^ ± 0.04	0.87 ^ab^ ± 0.25	4.73 ^a^ ± 0.34
WDH15%	4.38 ^a^ ± 0.23	2.21 ^a^ ± 0.36	−0.06 ^a^ ± 0.02	1.11 ^b^ ± 0.17	10.85 ^b^ ± 3.23
WDW5%	6.72 ^d^ ± 0.42	1.61 ^a^ ± 0.33	−0.04 ^a^ ± 0.00	0.84 ^a^ ± 0.02	8.89 ^b^ ± 1.50
WDW10%	5.93 ^bc^ ± 0.23	2.01 ^a^ ± 0.61	−0.04 ^a^ ± 0.00	0.82 ^a^ ± 0.09	9.69 ^b^ ± 2.29
WDW15%	4.14 ^a^ ± 0.03	2.07 ^a^ ± 0.66	−0.04 ^a^ ± 0.01	1.00 ^ab^ ± 0.01	8.63 ^ab^ ± 2.88
Factor 1	*p =* 0.318	*p =* 0.318	*p =* 0.074	*p =* 0.473	*p =* 0.043
Factor 2	*p* < 0.001	*p =* 0.129	*p =* 0.789	*p =* 0.021	*p =* 0.072
F1 × F2	*p* = 0.291	*p* = 0.430	*p* = 0.989	*p* = 0.791	*p* = 0.021

Mean values from five repetitions ± SD. Values in columns followed by the same superscript letters do not significantly differ at a significance level of 0.05. Factor 1 is the type of nut flour; Factor 2 is the supplementation level.

## Data Availability

All the data is available within the manuscript.
